# Sex differences in clinical profile, left ventricular remodeling and cardiovascular outcomes among diabetic patients with heart failure and reduced ejection fraction: a cardiac-MRI-based study

**DOI:** 10.1186/s12933-024-02362-4

**Published:** 2024-07-22

**Authors:** Ke Shi, Ge Zhang, Hang Fu, Xue-Ming Li, Li Jiang, Yue Gao, Wen-Lei Qian, Li-Ting Shen, Hua-Yan Xu, Yuan Li, Ying-Kun Guo, Zhi-Gang Yang

**Affiliations:** 1 Department of Radiology, Functional and Molecular Imaging Key Laboratory of Sichuan Province, West China Hospital, Sichuan University, Chengdu, Sichuan China; 2 Laboratory of Cardiovascular Diseases, Regenerative Medicine Research Center, West China Hospital, Sichuan University, Chengdu, Sichuan China; 3 Department of Radiology, Key Laboratory of Birth Defects and Related Diseases of Women and Children of Ministry of Education, West China Second University Hospital, Sichuan University, Chengdu, Sichuan China

**Keywords:** Heart failure with reduced ejection fraction, Diabetes mellitus, Sex, Cardiac magnetic resonance imaging, Outcomes

## Abstract

**Background:**

Heart failure with reduced ejection fraction (HFrEF) is associated with a high rate of mortality and morbidity. Evidence has shown that sex differences may be an important contributor to phenotypic heterogeneity in patients with HFrEF. Although diabetes mellitus (DM) frequently coexists with HFrEF and results in a worse prognosis, there remains a need to identify sex-related differences in the characteristics and outcomes of this population. In this study, we aimed to investigate the between-sex differences in clinical profile, left ventricular (LV) remodeling, and cardiovascular risk factors and outcomes in patients with HFrEF concomitant with DM.

**Methods:**

A total of 273 patients with HFrEF concomitant with DM who underwent cardiac MRI were included in this study. Clinical characteristics, LV remodeling as assessed by cardiac MRI, and cardiovascular risk factors and outcomes were compared between sexes.

**Results:**

Women were older, leaner and prone to have anemia and hypoproteinemia but less likely to have ischemic etiology. Cardiac MRI revealed that despite similar LVEFs between the sexes, there was more LV concentric remodeling, less impaired global systolic peak strain in longitudinal and circumferential components and a decreased likelihood of late gadolinium enhancement presence in women than in men. During a median follow-up time of 34.6 months, women exhibited better overall survival than men did (log-rank P = 0.042). Multivariable Cox proportional hazards analysis indicated different risk factors for predicting outcomes between sexes, with hypertension [hazard ratio (HR) = 2.05, 95% confidence interval (CI) 1.05 to 4.85, P = 0.041] and hypoproteinemia (HR = 2.27, 95% CI 1.06 to 4.37, P = 0.039) serving as independent determinants of outcomes in women, whereas ischemic etiology (HR = 1.96, 95% CI 1.11 to 3.48, P = 0.021) and atrial fibrillation (HR = 1.86, 95% CI 1.02 to 3.41, P = 0.044) served as independent determinants of outcomes in men.

**Conclusions:**

Among patients with HFrEF concomitant with DM, women displayed different LV remodeling and risk factors and had better survival than men did. Sex-based phenotypic heterogeneity in patients with HFrEF in the context of DM should be addressed in clinical practice.

## Introduction

Heart failure with reduced ejection fraction (HFrEF) is a debilitating syndrome and confers significant mortality and morbidity burdens. The posthospitalization 5 year survival rate for patients with HFrEF is only 25% [1]. At present, HFrEF remains a major public health concern with high associated healthcare costs [2]. There is a growing recognition that this disease appears to be a heterogeneous entity with a diverse set of epidemiological and pathophysiological factors and manifestations [3]. Among the potential contributors, sex differences were revealed by several recent studies, which led to the acknowledgment of sex-specific HFrEF phenotypes [4–7]. Although the prevalence of HFrEF increases with age in both sexes, the clinical presentation, risk factors, and long-term outcomes associated with HFrEF significantly differ between men and women. It has been demonstrated that women with HFrEF are older at diagnosis, have relatively elevated natriuretic peptide levels, and live longer than men. Moreover, there are also sex differences in underlying diseases, with women being more likely to have nonischemic cardiomyopathy, while men are more likely to have ischemic cardiomyopathy [8]. Identification of these discrepancies is beneficial for better addressing diagnostic and treatment strategies for patients with this condition.

Among patients with HFrEF, the incidence of diabetes mellitus (DM) is up to 40%, which is substantially greater than that for adults in the general population [9]. Data from clinical registries and multicenter studies demonstrate that concomitant DM portends a high risk of adverse outcomes for HF, with the greatest incremental risk observed in patients with HFrEF [10]. Poor outcomes are the result of distinct pathophysiological pathways by which DM affects myocardial remodeling in patients with HFrEF [9, 11]. However, in patients with HFrEF concomitant with DM, sex-related differences in clinical presentation, cardiac remodeling phenotype, and cardiovascular outcomes are not fully understood, but this information is important for clinicians to be able to recognize high-risk subtypes of cardiac failure. Herein, we sought to characterize the differences between men and women in terms of clinical features, diabetic left ventricular (LV) remodeling, and the impact on outcomes in patients with HFrEF.

## Methods

### Study population

In this study, patients with HFrEF who underwent cardiac MRI at our hospital between January 2015 and December 2022 were included. The diagnosis of HFrEF was made based on the guidelines from the European Society of Cardiology [12]. All patients met the following criteria: (1) had at least one symptom and/or sign of decompensated HF in the previous 3 months; (2) had a reduced (< 40%) LV ejection fraction (LVEF) as assessed by cardiac MRI; and (3) had an elevated amino-terminal pro-B-type natriuretic peptide (NT-proBNP) level. Patients who met at least one of the following criteria were excluded: (1) age younger than 18 years, (2) acute coronary syndrome, (3) severe arrhythmia, or (4) incomplete clinical or MRI information. DM status was defined as self-reported DM, current use of oral glucose-lowering medications, a fasting plasma glucose level higher than 7.0 mmol/L, or a hemoglobin A1c level greater than 6.5% [10]. At our institution, the reference range for albumin assays was 35–47 g/L, and the diagnosis of hypoproteinemia was made when the albumin concentration was less than 35 g/L. Anemia was diagnosed using the World Health Organization criteria: a hemoglobin concentration less than 120 g/L in nonpregnant adult females and less than 130 g/L in adult males [13].

Baseline data on demographics, clinical characteristics, laboratory measurements and medical treatments were retrieved from hospital records. This study was approved by the Biomedical Research Ethics Committees of our hospital and complied with the Declaration of Helsinki. All medical data were protected with full confidentiality and used only for the purpose of the present study.

### Imaging acquisition and postprocessing

Cardiac MRI was performed on a 3-Tesla scanner (MAGNETOM Skyra/Tim Trio; Siemens Healthcare, Erlangen, Germany) for each patient. Cine images were obtained during breath-holding at end-expiration using a balanced steady-state free precession (SSFP) sequence [repetition time (TR) = 2.81 ms; time to echo (TE) = 1.22 ms; slice thickness = 8.0 mm; flip angle (FA) = 40°/50°; acquisition matrix = 166 × 208 pixels; and field of view (FOV) = 340 × 284 mm^2^]. Approximately 10–15 short-axis images from the base to the apex were obtained, as well as 4-, 2- and 3-chamber long-axis images. Fifteen minutes after the administration of gadolinium-based contrast agent (0.2 mL/kg), late gadolinium enhancement (LGE) imaging was performed via a phase-sensitive inversion recovery sequence. The acquisition parameters were as follows: TR = 700/500 ms; TE = 1.18/1.07 ms; slice thickness = 8.0 mm; FA = 40°; acquisition matrix = 184 × 256 pixels; and FOV = 350 × 295 mm^2^.

All images were analyzed using commercially available CVI^42^ software (Circle Cardiovascular Imaging, Inc., Calgary, Alberta, Canada). For LV volumetric analyses, endocardial and epicardial borders were traced semiautomatically at the LV end-diastolic and end-systolic phases on the short-axis stacks and manually corrected if needed. LV volume and functional parameters, including EF, end-diastolic volume (EDV), end-systolic volume (ESV), and stroke volume (SV), were automatically calculated. LV papillary muscles were included in the LV mass (LVM) measurements but not in the LV volume measurements. LV volumetric measurements and LVM were indexed for body surface area. LV hypertrophy (LVH) was defined as an indexed LVM > 115 g/m^2^ in men and > 95 g/m^2^ in women. The LV remodeling index (LVRI) was calculated as the ratio of the LVM divided by the LVEDV. For LV contractility analyses, a stack of short-axis cine images combined with 4-, 2- and 3-chamber long-axis images were loaded into the feature-tracking module. We delineated the LV endocardial and epicardial borders at the end-diastolic phase (reference phase) of all cine images. The software automatically traced the contours throughout the cardiac cycle. Global myocardial peak strains in longitudinal (GLS), circumferential (GCS) and radial (GRS) components were calculated as the total deformation of the myocardium from its initial length at the end-diastolic phase to its final length at the end-systolic phase and are expressed as a percentage. Positive and negative signs of myocardial strain [peak strain (PS)] indicate shortening and thickening of the myocardium, respectively. The LV end-diastolic dimension (LVEDD) was assessed in the 4-chamber long-axis images. LGE assessment was performed by using the established grayscale threshold method, with those signal intensities exceeding six standard deviations (SDs) of remote nonfibrotic myocardium considered to indicate the presence of LGE.

### Cardiovascular outcome measures

The primary endpoint was the composite of HF hospitalization, cardiovascular mortality and heart transplantation, whichever occurred first. By reviewing electronic medical records or telephone interviews, we retrospectively collected follow-up data for all subjects until the occurrence of any endpoint or until censoring on December 31, 2023. The duration of follow-up was calculated as the time from undergoing cardiac MRI to either the occurrence of any endpoint or the last follow-up date.

### Statistical methods

Statistical analyses were performed using SPSS (IBM SPSS, Inc., Armonk, New York, USA) and Prism (GraphPad Software, Inc., San Diego, California, USA) software. The normality of the data was determined using the Shapiro–Wilk test. Continuous variables are presented as the means and SDs or medians and interquartile ranges (IQRs). Categorical variables are presented as counts and percentages. Between-group differences in baseline characteristics and cardiac MRI findings were examined using Student’s t test, the Wilcoxon–Mann–Whitney test, or the chi-square test (Fisher’s exact test), as appropriate. Long-term outcomes were assessed using Kaplan–Meier survival analysis and compared by sex with the log-rank test. To identify sex-related differences in cardiovascular risk factors in patients with HFrEF, a Cox proportional hazards model was used for the entire cohort and for men and women separately to determine the independent predictors associated with adverse outcomes. Each significant variable in the univariable analysis (P < 0.10) was included as a cofactor in the multivariable Cox proportional hazards model. A two-tailed P value < 0.05 was considered to indicate statistical significance.

## Results

### Baseline characteristics according to sex

In total, two hundred and seventy-three DM patients with HFrEF were ultimately included in this study, of whom 196 (71.8%) were men and 77 (28.2%) were women. The baseline characteristics by sex are shown in Table 1. On average, women were approximately 4.0 years older at diagnosis (P = 0.008) and had a lower body mass index (BMI) (P < 0.001) than men. There was a nonsignificant trend toward greater systolic blood pressure in women than in men (P = 0.089). In addition to DM, comorbidities were common in our study cohort; coronary artery disease was more prevalent among men (43.4% vs. 28.6; P = 0.024), while women were more likely to have anemia (20.4% vs. 35.1%; P = 0.011). In addition, there was a trend toward a greater prevalence of hypoproteinemia in women than in men (32.7% vs. 44.2%; P = 0.075). However, men and women displayed a similar burden of hypertension (HT), atrial fibrillation (AF), complete left bundle branch block and chronic obstructive pulmonary disease.

**Table 1:** Baseline characteristics of the study population by sex

	Men (n = 196)	Women (n = 77)	P-value
Age, years	55.2 ± 11.7	59.3 ± 10.4	0.008
BMI, kg/m^2^	25.2 ± 3.7	23.3 ± 3.4	< 0.001
SBP, mmHg	119.3 ± 19.6	124.0 ± 22.7	0.089
DBP, mmHg	79.5 ± 15.8	78.3 ± 13.4	0.595
HR, beats/min	87.1 ± 17.9	85.8 ± 16.7	0.601
NYHA functional class III– IV, n (%)	168 (85.7)	68 (88.3)	0.573
HF duration, n (%)			
≤ 1 year	106 (54.1)	42 (54.5)	0.958
> 1 and ≤ 5 years	59 (30.1)	22 (28.6)	
> 5 years	31 (15.8)	13 (16.9)	
Ischemic etiology, n (%)	66 (33.7)	16 (20.8)	0.036
Major comorbid conditions apart from DM, n (%)			
HT	97 (49.5)	35 (45.5)	0.548
CAD	85 (43.4)	22 (28.6)	0.024
AF	43 (21.9)	12 (15.6)	0.239
LBBB	13 (6.6)	8 (10.4)	0.295
COPD	18 (9.2)	4 (5.2)	0.276
Anemia, n (%)	40 (20.4)	27 (35.1)	0.011
Hypoproteinemia, n (%)	64 (32.7)	34 (44.2)	0.075
Laboratory measurements			
NT‑proBNP, pg/mL	2039 (1099, 4638)	2473 (1138, 5150)	0.628
TPN-T, ng/L	30.3 (19.9, 61.0)	24.4 (12.8, 52.8)	0.069
FBG, mmol/L	7.6 (6.2, 9.5)	7.9 (6.2, 10.1)	0.311
HbA1c, %	7.6 ± 1.8	7.3 ± 1.5	0.243
eGFR, mL/min/1.73m^2^	73.1 (58.3, 92.4)	73.2 (54.8, 92.7)	0.526
Hemoglobin, g/L	143.7 ± 23.5	126.1 ± 22.5	< 0.001
Albumin, g/L	40.7 ± 4.9	38.1 ± 5.1	0.096
Cardiovascular medications, n (%)			
Beta-blocker	157 (80.1)	56 (72.7)	0.185
ACEI/ARB	142 (72.4)	54 (70.1)	0.702
SGLT-2i	77 (39.3)	21 (27.3)	0.063
Loop diuretics	152 (77.6)	56 (72.7)	0.400
MRA	153 (78.1)	51 (66.2)	0.043
ARNI	108 (55.1)	35 (45.5)	0.151
CCB	26 (13.3)	11 (14.3)	0.825
Anti-thrombotic agents	114 (58.2)	36 (46.8)	0.088
Statins	101 (51.5)	32 (41.6)	0.138
Digoxin	33 (16.8)	18 (23.4)	0.212
Hypoglycemic medications, n (%)			
Insulin	62 (31.6)	26 (33.8)	0.734
Metformin	61 (31.1)	28 (36.4)	0.406
Sulfonylureas	26 (13.3)	12 (15.6)	0.618
α-GI	52 (26.5)	20 (26.0)	0.925
Diet control	39 (19.9)	11 (14.3)	0.281

Data are presented as mean ± SD, media (Q1, Q3) or number (percentage)

*BMI* body mass index, *SBP* systolic blood pressure, *DBP* diastolic blood pressure, *HR* heart rate, *NYHA* New York Heart Association, *HF* heart failure, *DM* diabetes mellitus, *HT* hypertension, *CAD* coronary artery disease, *AF* atrial fibrillation, *LBBB* complete left bundle branch block, *COPD* chronic obstructive pulmonary disease, *NT-proBNP* amino-terminal pro-B-type natriuretic peptide, *TPN-T* Troponin T, *FBG* fasting blood glucose, *HbA1c* glycated hemoglobin, *eGFR* estimated glomerular filtration rate, *ACEI* angiotensin converting enzyme inhibitor, *ARB* angiotensin receptor blocker, *SGLT-2i* sodium-glucose cotransporter-2 inhibitors, *MRA* mineralocorticoid receptor antagonist, *ARNI* angiotensin receptor-neprilysin inhibitor, *CCB* calcium-channel blocker, *α-GI* α-Glucosidase inhibitors

Women had a lower mean hemoglobin level (143.7 ± 23.5 g/L vs. 126.1 ± 22.5 g/L; P < 0.001) than men did, but the median concentrations of NT-proBNP, fasting blood glucose, and glycated hemoglobin and the estimated glomerular filtration rate (eGFR) did not significantly differ by sex (all P > 0.05). Women also had slightly but nonsignificantly lower albumin (40.7 ± 4.9 g/L vs. 38.1 ± 5.1 g/L; P = 0.096) and troponin T levels than men did [30.3 (19.9, 61.0) ng/L vs. 24.4 (12.8, 52.8) ng/L; P = 0.069]. There were no significant between-sex differences in the use of medications, except for mineralocorticoid receptor antagonists (P = 0.043).

### Sex differences in LV remodeling

**Fig. 1:**
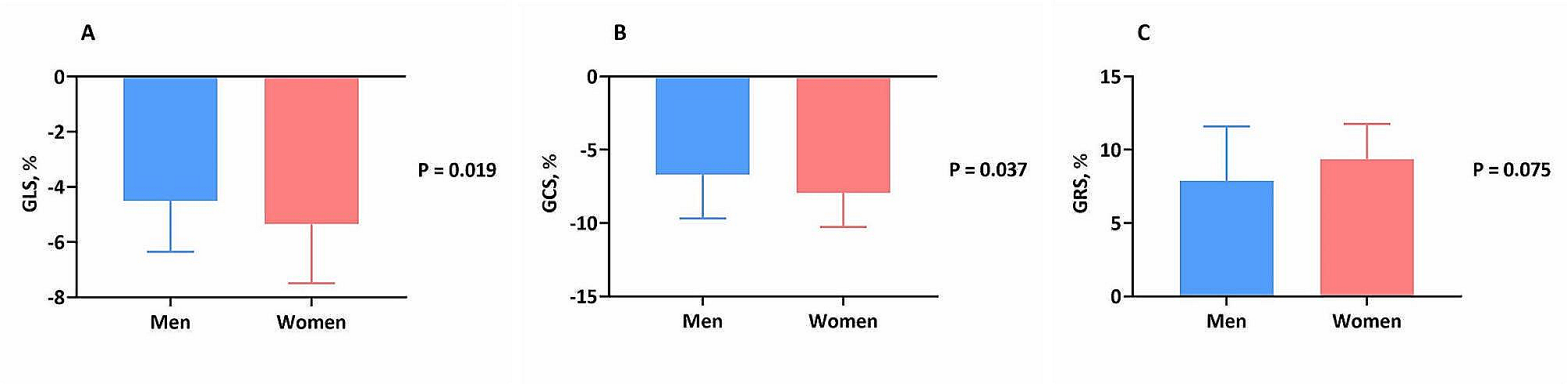
Sex differences in the magnitude of global LV systolic PS. *LV* left ventricular, *PS* peak strain

**Fig. 2:**
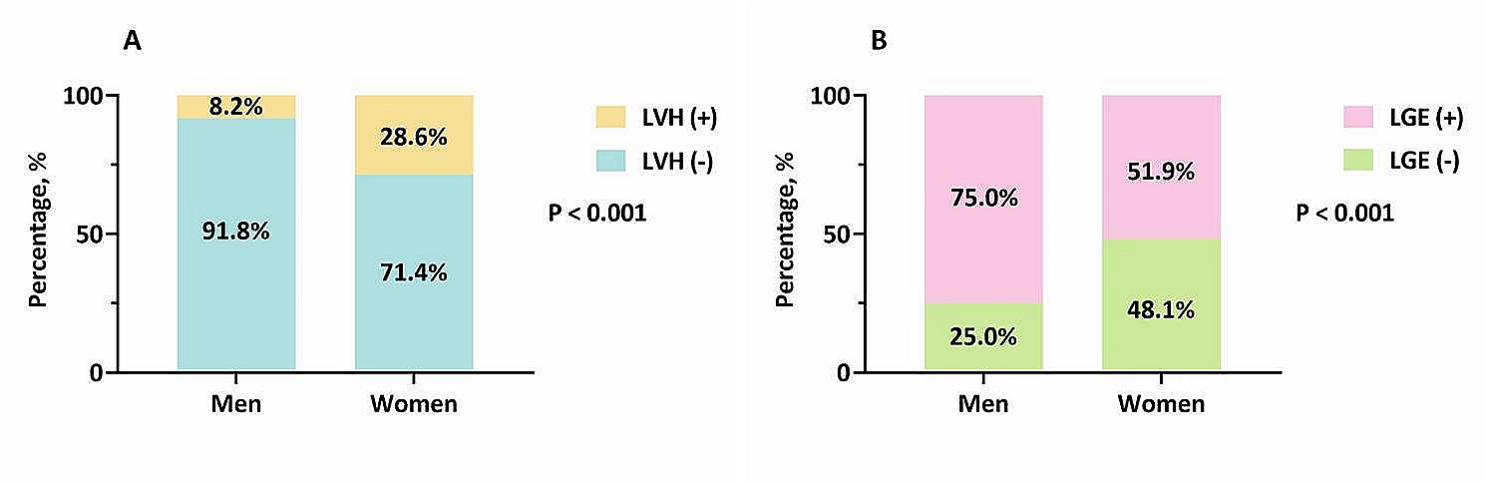
Sex differences in LVH and LGE presence. *LVH* left ventricular hypertrophy, *LGE* late gadolinium enhancement

Cardiac MRI revealed substantial between-sex differences, as detailed in Table 2. Men had greater LVEDV index [152.4 (121.3, 185.3) mL/m^2^ vs. 137.3 (110.2, 168.6) mL/m^2^; P = 0.009], LVESV index [115.9 (85.2, 147.6) mL/m^2^ vs. 101.7 (76.5, 126.1) mL/m^2^; P = 0.017] and LVEDD (63.7 ± 12.0 mm vs. 56.7 ± 10.9 mm; P < 0.001) than women did. Although the LVEF was comparable between men and women, more severe decreases in the GLS [− 4.5% (− 3.3%, − 6.3%) vs. − 5.4% (− 3.8%, − 7.5%); P = 0.019] and GCS [– 6.7% (− 4.8%, − 9.7% vs. − 8.0% (− 5.4%, − 10.3%); P = 0.037] were noted in men than in women. Moreover, there was a trend toward a lower magnitude of GRS [7.8% (5.3%, 11.6) vs. 9.4% (6.3%, 11.8%); P = 0.075] in men than in women (Fig. 1). Despite no significant between-sex differences in the LVM index, compared to women, men had a lower LVRI [0.5 (0.4, 0.6) vs. 0.6 (0.5, 0.8); P = 0.004], accompanied by a lower prevalence of LVH (8.2% vs. 28.6; P < 0.001) and a higher prevalence of LGE (75.0% vs. 51.9%; P < 0.001) (Fig. 2).

**Table 2:** Cardiac MRI findings according to sex

	Men (n = 196)	Women (n = 77)	P-value
LVEDV index, mL/m^2^	152.4 (121.3, 185.3)	137.3 (110.2, 168.6)	0.009
LVESV index, mL/m^2^	115.9 (85.2, 147.6)	101.7 (76.5, 126.1)	0.017
LVSV index, mL/m^2^	34.6 (27.1, 45.3)	31.7 (24.7, 42.0)	0.128
LVEF, %	24.1 (17.0, 31.5)	27.5 (19.9, 32.5)	0.200
LVEDD, mm	63.7 ± 12.0	56.7 ± 10.9	< 0.001
LVM index, g/m^2^	81.4 ± 18.7	81.9 ± 18.2	0.832
LVRI	0.5 (0.4, 0.6)	0.6 (0.5, 0.8)	0.004
LVH, n (%)	16 (8.2)	22 (28.6)	< 0.001
LGE, n (%)	147 (75.0)	40 (51.9)	< 0.001

Data are presented as mean ± SD, media (Q1, Q3) or number (percentage)

*LVEDV* left ventricular end-diastolic volume, *LVESV* left ventricular end-systolic volume, *LVSV* left ventricular stroke volume, *LVEF* left ventricular ejection fraction, *LVEDD* left ventricular end-diastolic dimension, *LVM* left ventricular mass, *LVRI* left ventricular remodeling index, *LVH* left ventricular hypertrophy, *LGE* late gadolinium enhancement

### Sex differences in cardiovascular outcomes

**Fig. 3:**
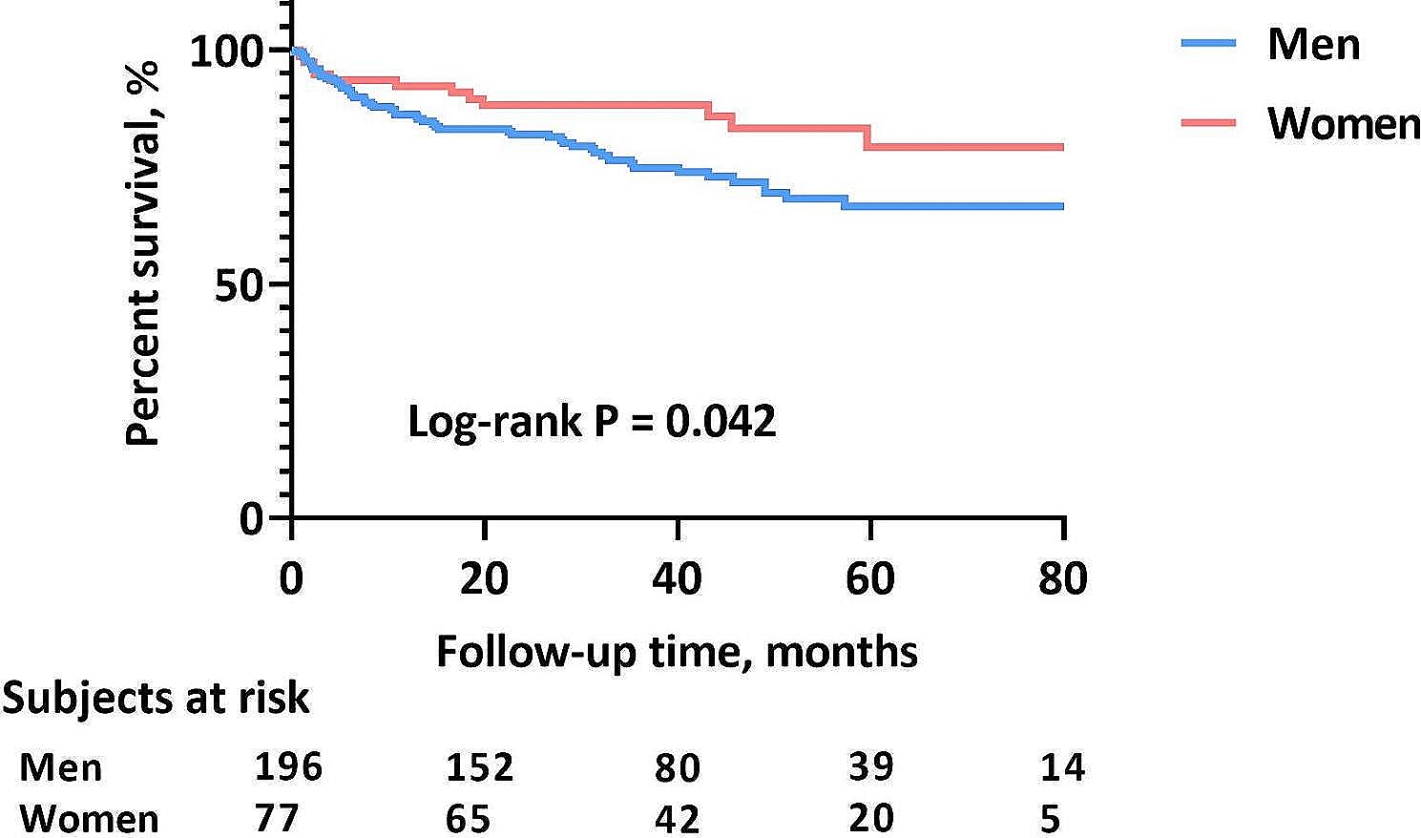
Between-sex comparison of OS rates. *OS* overall survival

During a median follow-up time of 34.6 (IQR, 21.7, 56.9) months, a total of 71 patients (26.0%) experienced at least one confirmed adverse outcome, of whom 58 patients (21.2%) were hospitalized due to HF progression, 8 patients (2.9%) died and 5 patients (1.8%) underwent heart transplantation. Kaplan–Meier survival analysis revealed better overall survival (OS) in women than in men (log-rank P = 0.042) (Fig. 3).

In a univariate Cox proportional hazards model involving the entire study cohort, ischemic etiology, HT, anemia, insulin use, LGE presence and GLS were found to be predictors of cardiovascular outcomes, in addition to age, BMI, NT-proBNP, eGFR and LVEF, which are established clinical variables with prognostic profiles (data not shown). In a multivariable Cox proportional hazards model incorporating age, BMI, NT-proBNP, eGFR and LVEF, ischemic etiology, HT, anemia, insulin use, LGE presence and GLS were separately associated with adverse outcomes. The details are displayed in Table 3.

**Table 3:** Cox proportional hazards regression model to identify associated variables of cardiovascular outcomes

	Univariable analysis		Multivariable analysis^*^	
	HR (95% CI)	P-value	HR (95% CI)	P-value
Ischemic etiology	1.87 (1.17, 3.00)	0.010	1.85 (1.12, 3.05)	0.016
HT	2.04 (1.27, 3.29)	0.003	1.97 (1.18, 3.29)	0.010
AF	1.63 (0.98, 2.71)	0.061	1.60 (0.94, 2.73)	0.084
Anemia	2.07 (1.29, 3.33)	0.003	2.22 (1.29, 3.84)	0.004
Hypoproteinemia	1.49 (0.93, 2.38)	0.099	1.49 (0.88, 2.51)	0.138
Insulin use	2.12 (1.33, 3.38)	0.002	2.03 (1.23, 3.33)	0.005
LGE present	2.54 (1.41, 3.57)	0.002	2.74 (1.46, 4.14)	0.002
GLS^#^	1.16 (1.04, 1.30)	0.010	1.27 (1.09, 1.43)	0.002

*HR* hazards ratio, *CI* confidence interval, *HT* hypertension, *AF* atrial fibrillation, *LGE* late gadolinium enhancement, *GLS* global longitudinal peak strain, *BMI* body mass index; *NT-proBNP* amino-terminal pro-B-type natriuretic peptide, *eGFR* estimated glomerular filtration rate, *LVEF* left ventricular ejection fraction

^*^Multivariable model is built after adjusting for age, BMI, NT‑proBNP, eGFR and LVEF. Each listed variable enters this model separately

^#^GLS is negative value in this analysis

**Fig. 4:**
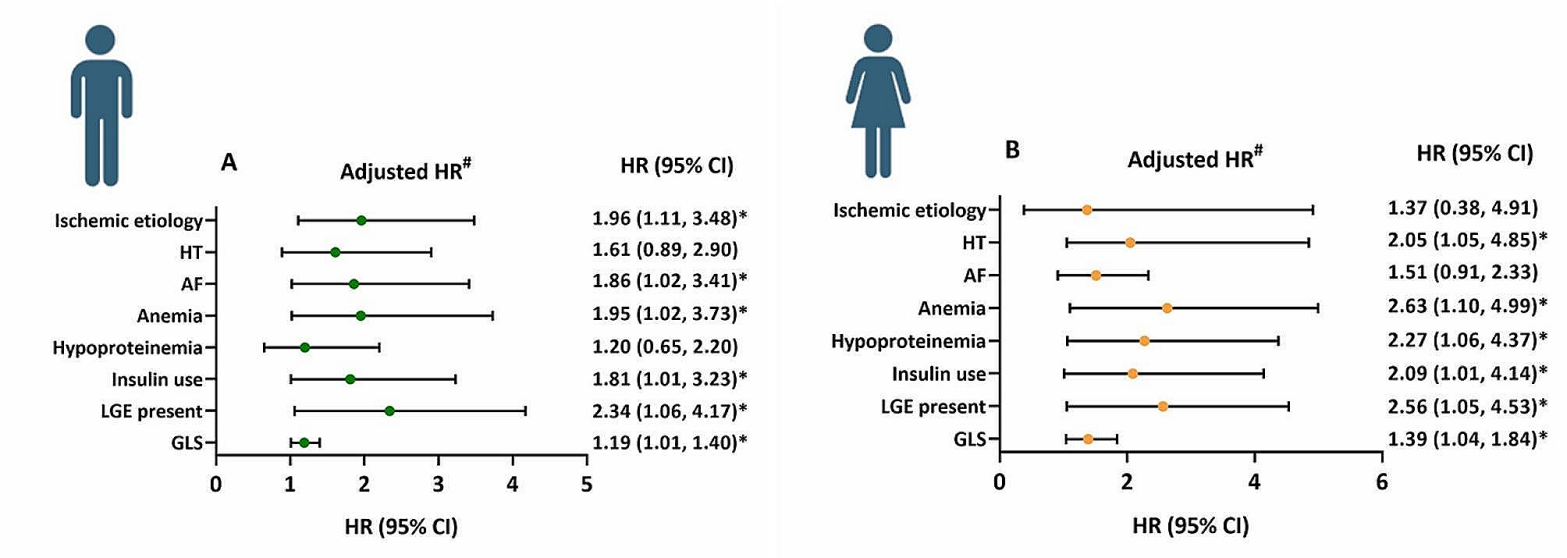
Differences in the risk of cardiovascular outcomes between men (**A**) and women (**B**). ^#^The hazard ratio for each variable was calculated separately using a multivariable model adjusted for age, BMI, NT-proBNP, eGFR and LVEF, which are clinically confirmed variables with prognostic utility, GLS is included in this model with negative value. *BMI* body mass index, *NT-proBNP* amino-terminal pro-B-type natriuretic peptide; *eGFR* estimated glomerular filtration rate, *LVEF* left ventricular ejection fraction, *HT* hypertension, *AF* atrial fibrillation, *LGE* late gadolinium enhancement, *GLS* global longitudinal strain, *HR* hazard ratio, *CI* confidence interval

When considering factors affecting the achievement of the composite outcomes in men and women separately, there were different predictors of outcomes. As depicted in Fig. 4, anemia [men: hazard ratio (HR) = 1.96, 95% confidence interval (CI) = 1.02 to 3.73, P = 0.043; women: HR = 2.63, 95% CI = 1.10 to 4.99, P = 0.035], insulin use [men: HR = 1.81, 95% CI 1.01 to 3.23, 0.045; women: HR = 2.09, 95% CI 1.01 to 4.14, P = 0.048], LGE presence [men: HR = 2.34, 95% CI 1.06 to 4.17, P = 0.036; women: HR = 2.56, 95% CI 1.05 to 4.53, P = 0.041] and GLS [men: HR = 1.19, 95% CI 1.01 to 1.40, P = 0.033; women: HR = 1.39, 95% CI 1.04 to 1.84, P = 0.026] were the common independent predictors of adverse outcomes for both men and women. Notably, ischemic etiology (HR = 1.96, 95% CI = 1.11 to 3.48, P = 0.021) and AF (HR = 1.86, 95% CI 1.02 to 3.41, P = 0.044) were independently associated with poor outcomes in men. In contrast, HT (HR = 2.05, 95% CI  1.05 to 4.85, P = 0.041) and hypoproteinemia (HR = 2.27, 95% CI  1.06 to 4.37, P = 0.039) served as the other significant outcome predictors in women.

## Discussion

With respect to patients with HFrEF concomitant with DM, there were several findings from the current study: (1) Women accounted for only a minority of the study cohort, and they were older and leaner than the included men and were less likely to have an ischemic etiology. Nevertheless, anemia and hypoproteinemia appeared to be more common in women than in men. (2) Despite similar LVEFs between sexes, women had smaller LV dimensions and volumes, even after adjusting for body size. Women were prone to LV concentric remodeling, accompanied by less impaired global myocardial systolic PS and a lower likelihood of LGE presence. (3) Women had better OS than men. After adjustment for covariates, differentiated risk factors for prognosis were identified between sexes, with HT and hypoproteinemia serving as independent predictors of outcomes in women and ischemic etiology and AF in men and anemia in both sexes.

### Sex differences in clinical profiles

In this study, we compared the between-sex differences in clinical characteristics in patients with HFrEF concomitant with DM. Similar to prior studies focused on sex differences in patients with HFrEF, but irrespective of DM status, we found that women with HFrEF concomitant with DM were older than men at the time of referral [5, 6]. However, in the present study population, a lower BMI was observed among women. One reasonable explanation for this observation may be racial/ethnic differences, which indicates that Asian patients with DM tend to be leaner than their Western counterparts [14]. As shown in the present study, existing DM did not modify the relationship between the ischemic etiology of HF and sex. In contrast, DM itself could accelerate the progression of coronary atherosclerosis, causing a greater burden and earlier onset of CAD in men than in women [4, 15]. Furthermore, despite the similar physician-assessed severity of HF (NYHA functional class) and plasma concentrations of NT-proBNP in our study between men and women, the prevalence of anemia was much greater in women than in men. Similarly, there was also a trend toward a greater proportion of patients with hypoproteinemia among women than among men. This may suggest that women with HFrEF concomitant with DM are likely to have a poor nutritional status, which is followed by more symptoms and signs of HF (e.g., edema, congestion). Thus, the present study could provide further evidence to support the previous finding that compared with men, women with HFrEF have worse quality of life [5, 7, 16].

### Sex differences in LV remodeling

To our knowledge, the current study is the first to assess sex differences in diabetic LV remodeling in patients with HFrEF using cardiac MRI. A previous echocardiography-based study showed that patients with HFrEF concomitant with DM had smaller LV volumes than their non-DM counterparts did [17]. The results from our study extended the findings to further reveal the fundamental differences between men and women, demonstrating a smaller LV size and volume in women; in addition, compared to men, women were inclined to present with concentric remodeling. Since it is conceivable that eccentric remodeling and LV dilatation predominate in patients with HFrEF, increased extracellular matrix deposition of collagen glycated end products in response to disturbance of glucose metabolism exerted by DM cannot fully explain the sex discrepancy in LV geometry observed in our study [18]. However, some evidence suggests that women tend to present with more pronounced LVH due to their susceptibility to augmented LV load pressure [19]. In addition, the age-dependent increase in LV wall thickness may also play a role in sex differences in the cardiac remodeling process [20].

To date, few studies have examined between-sex differences in scar formation and myocardial mechanics among patients with HFrEF concomitant with DM. We observed a greater rate of detectable LGE and more severe deterioration of LV systolic function, as measured by myocardial strain analysis, in men than in women. In terms of this observation, updated evidence indicates a specific myocardial injury driven by cardiomyocyte cell death in patients with HFrEF, and concomitant DM aggravates this process through lipotoxicity or advanced glycation end products, particularly in the male heart, which is susceptible to the ischemic pattern of focal fibrosis [9, 11, 21]. In addition, sex hormones, which can exert a cardioprotective effect on the female heart, are considered another factor responsible for the differences in LV fibrosis detected using LGE [22]. In such a case, the LV systolic function in women could be retained. However, further studies are needed to determine the underlying mechanisms responsible for the between-sex differences in diabetes-related cardiac remodeling.

### Sex differences in cardiovascular outcomes

With respect to outcomes, our data showed that women with HFrEF concomitant with DM had a lower rate of the composite endpoint than men did. This finding was in line with several prior studies focused on overall HFrEF cohorts [4–7]. Notably, a previous study by Chandramouli et al. revealed an inverse relationship between sex and prognosis, with poor outcomes exhibited in diabetic women with HFrEF [19]. However, in contrast to the findings from our study, their observations were based on a one-year follow-up. Furthermore, nearly one-third of the participants in this study were from low-income regions, and a relatively greater burden of comorbidities may have contributed to the observed differences in prognosis.

Interestingly, in addition to confirming the prognostic utility of GLS, LGE presence and insulin use that has already been established previously, our study revealed disparate impacts of cardiovascular risk factors on prognosis among men and women with HFrEF concomitant with DM. Since ischemic heart disease predominates as a risk factor in men and can be exacerbated by DM, it is not surprising that ischemic etiology is independently related to adverse outcomes in men. Furthermore, in diabetic men, our data indicated that the subsequent development of AF was associated with an increased risk of adverse outcomes in patients with HFrEF. A study based on a nationwide registry revealed that among individuals with HFrEF, AF affects more men than women [23]. In healthy individuals, a larger left atrial volume, which is intrinsically predisposed to AF, has been observed in men [24]. Given the observed LV remodeling with chamber dilatation in our study, we speculate that left atrial enlargement occurring in response to elevated left-side pressure together with atrial fibrosis may be a representation of underlying pathologies [25–27].

Nevertheless, our findings differ from those of a previous report suggesting that anemia was associated with myocardial injury in men but not in women. The authors attributed this finding to younger age in women with a low prevalence of anemia and a predominance of anemia-related oxidative stress in men [28]. However, in the current study, which focused on HFrEF, women were more likely to suffer from anemia, perhaps due to their older age. Moreover, the presence of DM in our cohort exerted a synergistic effect on oxidative stress, which could induce myocardial injury and thereby lead to anemia, which is a common risk factor in both sexes [29]. In the Framingham Heart Study, hypertensive women had a greater population-attributable risk of HF, regardless of their similar prevalence of HT. It has been revealed that women with HT concomitant with DM often have worse blood pressure control, and HT-mediated organ damage is more obvious in the presence of DM, which may explain why HT is independently associated with adverse outcomes in women but not in men [8, 10, 30]. Finally, the plasma concentration of albumin is influenced mainly by hepatic synthesis, exogenous intake and loss. A recently published article highlighted the key role of albumin in the survival of patients with HFrEF [31]. Our finding that hypoproteinemia is associated with poor outcomes in women but not in men in the context of HFrEF concomitant with DM implies a potential causal role between liver dysfunction and hypoproteinemia. Whether diabetic women with HFrEF have elevated right atrial pressure and hepatic venous congestion remains unclear [32, 33]. Moreover, systemic inflammation and sympathetic activity in diabetic women may be considered other contributors [34].

## Study limitations

We must acknowledge several limitations in this study. First, due to a relatively smaller sample sizes and fewer outcomes in women (although this is very common in studies on HFrEF), we used the composite outcome to maximize the statistical power. Further studies with expanded study cohorts are encouraged to explore sex-specific differences in each adverse outcome. Second, patient-reported HF severity, such as that indicated by the Kansas City Cardiomyopathy Questionnaire score, and symptoms or signs related to HF were not assessed in our study. It would be interesting to clarify sex differences in the role of DM in psychological and physical disability among patients with HFrEF. Third, given the long-time span in this study (from January 2015 to December 2022), a considerable proportion of patients didn’t receive T1 mapping and extracellular volume fraction evaluations for the limited technical conditions several years ago. So, we didn’t have enough data to analysis LV tissue characterization (e.g. interstitial fibrosis) in our patients by using this promising method. Last, this was an observational study that was carried out on a retrospective HF cohort. Thus, selection bias is inevitable.

## Conclusions

In conclusion, the present study highlights the evidence supporting sex-based differences in clinical profile, LV remodeling, and prognosis. Although women with HFrEF concomitant with DM exhibited better postdiagnosis survival than men did, they had significantly different cardiovascular risk factors than men who experienced adverse events. Our findings reinforce the notion that sex may contribute to the phenotypic heterogeneity of patients with HFrEF concomitant with DM, which provides a basis for clinicians to address sex-specific differences in diagnosis, risk factor management, and the implementation of treatments that improve prognosis.

## Data Availability

No datasets were generated or analysed during the current study.

## Abbreviations

HFrEF: Heart failure with reduced ejection fraction
T2DM: Type 2 diabetes mellitus
LV: Left ventricular
MRI: Magnetic resonance imaging
LVEF: Left ventricular ejection fraction
NT-proBNP: Amino-terminal pro-B-type natriuretic peptide
SSFP: Steady-state free precession
TR: Repetition time
TE: Time to echo
FA: Flip angle
FOV: Field of view
LGE: Late gadolinium enhancement
EDV: End-diastolic volume
ESV: End-systolic volume
SV: Stroke volume
LVM: Left ventricular mass
LVH: Left ventricular hypertrophy
GLS: Global longitudinal strain
GCS: Global circumferential strain
GRS: Global radial strain
PS: Peak strain
LVEDD: Left ventricular end-diastolic dimension
SDs: Standard deviations
IQRs: Interquartile ranges
HT: Hypertension
AF: Atrial fibrillation
eGFR: Estimated glomerular filtration rate
HR: Hazard ratio
CI: Confidence interval
